# Complete genome sequence of *Bacillus licheniformis* strain Ukraine01 isolated from bovine biomaterial in Ukraine

**DOI:** 10.1128/mra.00894-26

**Published:** 2026-10-13

**Authors:** Anton P. Gerilovych, Artem V. Ushkalov

**Affiliations:** 1 Institute for Problems of Cryobiology and Cryomedicine of NAS of Ukraine, Kharkiv, Ukraine; 2 One Health Scientific and Research Institute, PSI, Kharkiv, Ukraine; 3 National Scientific Center “Institute for Experimental and Clinical Veterinary Medicine”, Kharkiv, Ukraine; USDA-ARS San Joaquin Valley Agricultural Sciences Center, Parlier, California, USA

**Keywords:** *Bacillus licheniformis*, genome, NGS, sequencing, Ukraine

## Abstract

We report the complete genome sequence of *Bacillus licheniformis* strain Ukraine01, isolated from bovine biomaterial collected in Kharkiv, Ukraine, in 2024. This is one of the first publicly available complete genomes of a veterinary-associated *B. licheniformis* isolate from Ukraine, an agent of secondary infections and a valuable resource for epidemiological studies.

## ANNOUNCEMENT

*Bacillus licheniformis* is a gram-positive, aerobic or facultatively anaerobic, endospore-forming bacterium belonging to the genus *Bacillus* and family *Bacillaceae*. The species is ubiquitous in nature and commonly occurs in soil, plant material, water, feed, and animal-associated environments. *B. licheniformis* is able to produce numerous extracellular enzymes, antimicrobial peptides, and industrially important metabolites for biotechnological purposes. It is also recognized as an opportunistic pathogen causing mastitis, abortion, septicemia, wound infections, and food spoilage in animals and humans (1–4).

*Bacillus licheniformis* strain Ukraine01 (2024) was isolated from bovine biomaterial after 36 h of cultivation in lysogeny broth (LB) liquid medium at 37°C, and identified as *Bacillus licheniformis* based on its characteristic colony morphology, gram-positive rod-shaped cells, spore formation, and biochemical properties, including catalase activity and utilization of selected carbohydrates.

Genomic DNA was extracted using the MagnetPure DNA/RNA Kit (Ukraine) and sequenced on the Oxford Nanopore Technologies MinION platform using Rapid sequencing DNA V14 barcoding (SQK-RBK114.24) according to the manufacturer’s guidelines. Basecalling, consensus polishing, and assembly were performed using Epi2ME v5.2.5 with the microbial genomes workflow (5). The draft assembly was polished with Medaka v5.0.0 (6). Genome annotation was conducted using the DFAST annotation pipeline v 1.4.2 (7). Default parameters were used at these stages of the study.

The resulting assembly consists of a single circular chromosome measuring 4,333,232 bp. No plasmids were reported in the deposited genome. The complete annotated genome has been deposited in GenBank under accession AP044034.1 (Table 1).

**TABLE 1:** Assembly and annotation summary of *Bacillus licheniformis* strain Ukraine01

Parameter	Genome data
BioProject	PRJDB37411
BioSample	SAMD01620630
Sequence Read Archive	DRS772151
Genome assembly accession	AP044034.1
Organism	*Bacillus licheniformis* strain Ukraine01
Isolation source	*Bos taurus*
Collection location	Kharkiv, Ukraine
Sequencing platform	Oxford Nanopore Technologies MinION
Assembly software	Medaka v5.0.0 (Epi2ME workflow)
Genome coverage (×)	~100
Assembly size (bp)	4,333,232
Chromosomes	1 (circular)
Plasmids	None detected
No. of contigs	1
Read N50 (bp)	1,360
L50	1
Longest contig (bp)	4,333,232
Guanine-cytosine content (%)	45.8
Coding sequences (CDSs)	5,427
rRNA genes	24
tRNA genes	80
Annotation pipeline	DFAST
Total length (bp)	4,333,232
No. of sequences	1
Gap ratio (%)	0.0
Coding ratio (%)	84.0

Genome annotation identified numerous genes involved in essential cellular processes, including DNA replication, transcription, translation, amino acid biosynthesis, carbohydrate metabolism, transport systems, and sporulation. Conserved housekeeping genes such as *dnaA, dacA, tmk, pdxS, pdxT, darA, ricT,* and *bofA* were identified, together with multiple ribosomal RNA operons and transfer RNA genes characteristic of complete *Bacillus* genomes.

Genes associated with sporulation and environmental persistence are well represented, reflecting the ecological versatility of *B. licheniformis*.

**Fig 1:**
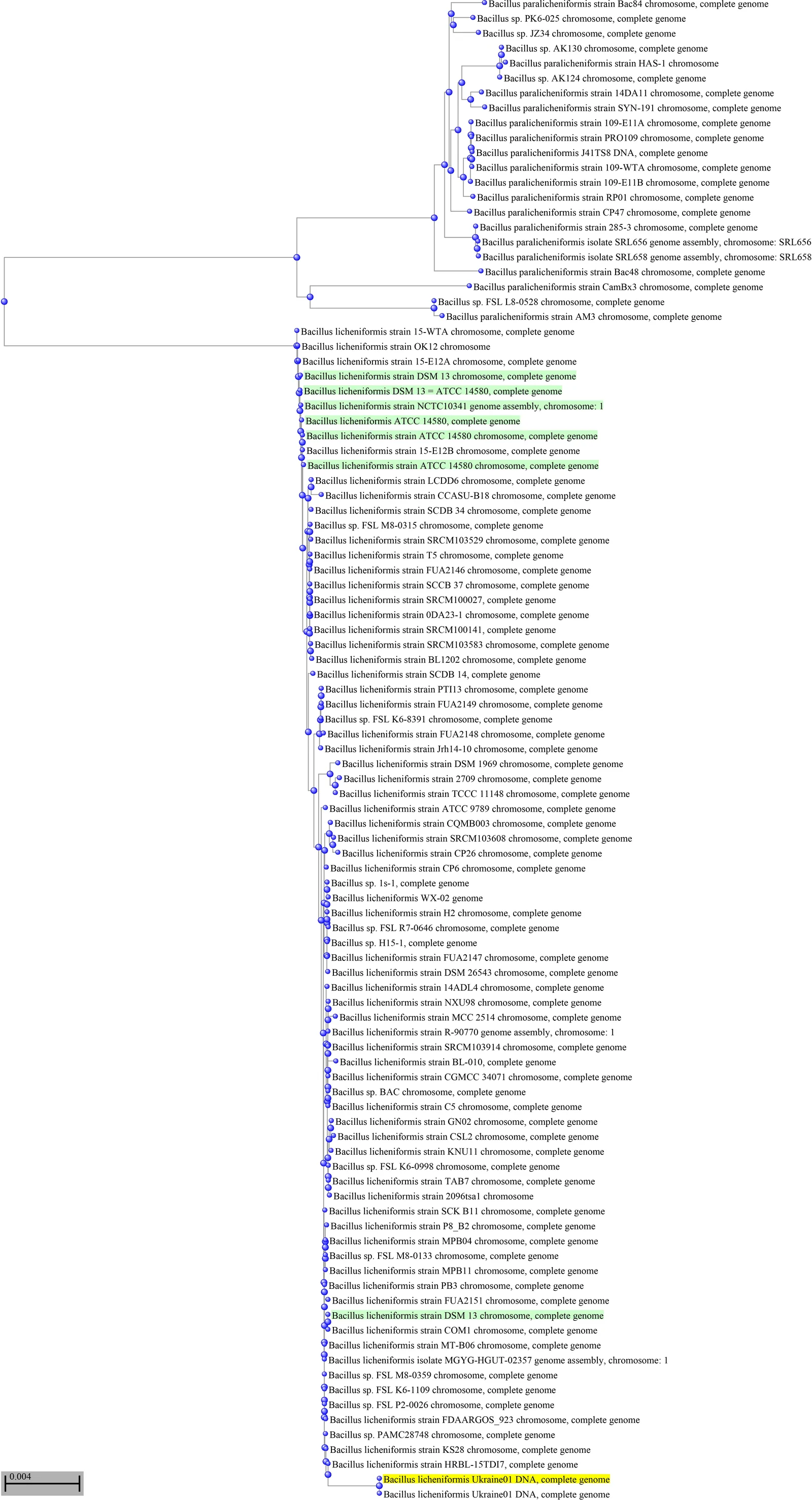
Minimum evolution phylogenetic tree of worldwide isolated *B. licheniformis* strains (Ukrainian isolate highlighted in yellow).

After comparing the assembled genome using the minimum evolution algorithm in the GenBank BLASTn module (1,000 bootstrap replicates), we determined that the closest relative of our isolate was represented by the strain HRBL-15TDI7 from South Korea (Fig. 1) (8).

As a complete chromosome assembly derived from long-read sequencing, the Ukraine01 genome provides a robust genomic reference for future comparative studies of veterinary *B. licheniformis* isolates. Availability of this genome will facilitate analyses of biology and evolutionary relationships among strains circulating in Europe and Asia.

## Data Availability

The complete genome sequence of *Bacillus licheniformis* strain Ukraine01 has been deposited in DDBJ/ENA/GenBank under accession AP044034.1 as part of BioProject PRJDB37411 and BioSample SAMD01620630, Assembling (GCA_055397075.1). Annotation and raw data workflow data available on https://dfast.ddbj.nig.ac.jp/analysis/annotation/7ca0ccd6-1478-4a77-b494-e257427863ae. Sequencing raw data (SRA) is available from NCBI DRS772151.
